# Diverse weaning foods and diet patterns at multiple time points during infancy period and their association with neurodevelopmental outcomes in 6-year-old children

**DOI:** 10.1038/s41430-024-01528-3

**Published:** 2024-10-18

**Authors:** Ju Hee Kim, Eun Kyo Ha, Gi Chun Lee, Boeun Han, Jeewon Shin, Man Yong Han, Seonkyeong Rhie

**Affiliations:** 1 Department of Pediatrics, College of Medicine, Kyung Hee University Medical Center, Kyung Hee University, Seoul, South Korea; 2 Department of Pediatrics, Hallym University Kangnam Sacred Heart Hospital, Hallym University College of Medicine, Seoul, South Korea; 3 Department of Computer Science and Engineering, College of Engineering, Konkuk University, Seoul, South Korea; 4 Department of Pediatrics, Bundang CHA Medical Center, CHA University School of Medicine, Seongnam, South Korea; 5 Department of Pediatrics, Ilsan CHA Medical Center, CHA University School of Medicine, Goyang, South Korea

**Keywords:** Paediatrics, Nutrition

## Abstract

**Background/Objectives:**

Understanding the impact of early-life nutritional choices on neurodevelopment in children is a growing area of research. To investigate the association between dietary patterns at multiple timelines and neurodevelopmental outcomes in 6-year-old children.

**Subjects/Methods:**

This administrative observational study utilized a merged data from the national health insurance database and the health screening program for children. Information on the diet patterns from infancy to 3 years of age was obtained from parent-administered questionnaires. Dietary pattern clusters of the participants were identified using Polytomous Latent Class Analysis. The outcome was neurodevelopment using the Korean Developmental Screening Test (K-DST) at the age of 6 years.

**Results:**

The study identified four distinct clusters among with the 133,243 eligible children (49.6% male, birth weight 3.22 kg, head circumference 42.7 cm at 4 months). The control cluster (53.4%) exhibited a diet including breast milk feeding and a variety of dietary patterns at the age of 1 year. In contrast, cluster 1 (36.0%) showed a skewed dietary pattern at the same age. Cluster 2 (6.6%) displayed diverse dietary patterns at one year but primarily consumed formula at four months, while cluster 3 (4.0%) had reduced dietary diversity and formula feeding. Compared with the control cluster, the adjusted odds ratio for unfavorable development was 1.209 (95% CI, 1.156–1.266) in cluster 1, 1.418 (95% CI, 1.312–1.532) in cluster 2, and 1.741 (95% CI, 1.593–1.903) in cluster 3. These findings remained consistent across individual domains of the K-DST.

**Conclusions:**

Dietary patterns during infancy and early childhood may be associated with neurodevelopment at the age of 6 years.

## Introduction

Neurodevelopmental growth and maturation occur rapidly during the fetal and infant periods. The brain exhibits a high degree of plasticity during this period [1]. Various factors influence cognitive development, including parent-child interaction, environmental complexity, parental socioeconomic status, nutritional support, and physical training [2–4]. The enriched environment has also been shown to impact neurogenesis, a factor affecting neural development significantly [5, 6]. An enriched environment includes various stimuli for physical activity, sufficient space, enough nutritional support, and physical training [7]. Additionally, enriched sensory and physical stimulation factors, such as nesting material, are also important [5, 8]. The mechanisms underlying the influence of enriched environments on brain development involve changes in the presentation of ion channels and neural circuits [9].

The type and variety of food can also play a role in creating an enriched environment. A restrictive diet is associated with early neurodevelopmental problems [10]. In an animal study, rats fed a soft diet showed negative effects on neurodevelopmental outcomes, particularly cell proliferation in the dentate gyrus of the hippocampus [11]. Similarly, a cross-sectional study conducted in China’s rural areas found that children who consumed a low number and variety of food items had delayed neurodevelopmental outcomes [12]. These findings demonstrate that feeding patterns during the neuro-vulnerable period of early infancy significantly influence neural development. Conversely, analyzing dietary patterns at a single time point during the postnatal period may pose challenges in evaluating developmental outcomes, as infant diets tend to change over time.

We hypothesize that dietary patterns during early childhood can influence neurodevelopment later in life. Therefore, this study aimed to investigate the effect of diverse weaning foods and diet patterns at multiple time points, utilizing clustering analysis utilizing cluster analysis and controlling for socioeconomic status, on children’s neurodevelopmental growth in six developmental domains. The findings from this study can be used to present appropriate guidelines for children’s eating habits to caregivers based on dietary patterns at various stages rather than at a single point in time.

## Methods

### Study design and data source

This study utilized a merged database from the National Health Insurance Service (NHIS) and the National Health Screening Program for Infants and Children (NHSPIC) in Korea. The NHIS is a single insurance system covering nearly the entire Korean population, making it a representative data source. The NHIS database provides baseline demographic characteristics, such as birth date, sex, insurance premium, and region of residence, as well as information on healthcare utilization, including the type of hospital visit, diagnosis codes (*International Classification of Diseases 10th revision* [ICD-10] codes), prescribed medication codes, and procedure codes. All children in Korea were eligible to undergo seven rounds of the NHSPIC, which were conducted at specific age intervals from four to 72 months of age. The rounds were scheduled as follows: 1st (4–6 months old), 2nd (9–12 months old), 3rd (18–24 months old), 4th (30–36 months old), 5th (42–48 months old), 6th (54–60 months old), and 7th (66–72 months old). The NHSPIC survey includes a general health questionnaire, the Korean Developmental Screening Test (K-DST), an anthropometric examination, and a physical examination [13].

The de-identified individual data were used only for research purposes. Patient consents were not required as this study was based on de-identified and publicly available data. The Institutional Review Board of the Korea National Institute for Bioethics Policy waived the need for informed consent. The study protocol was reviewed and approved by the Institutional Review Board of the Korea National Institute for Bioethics Policy (P01-201603-21-005). All methods were performed in accordance with the relevant guidelines and regulations.

### Study population

**Fig. 1:**
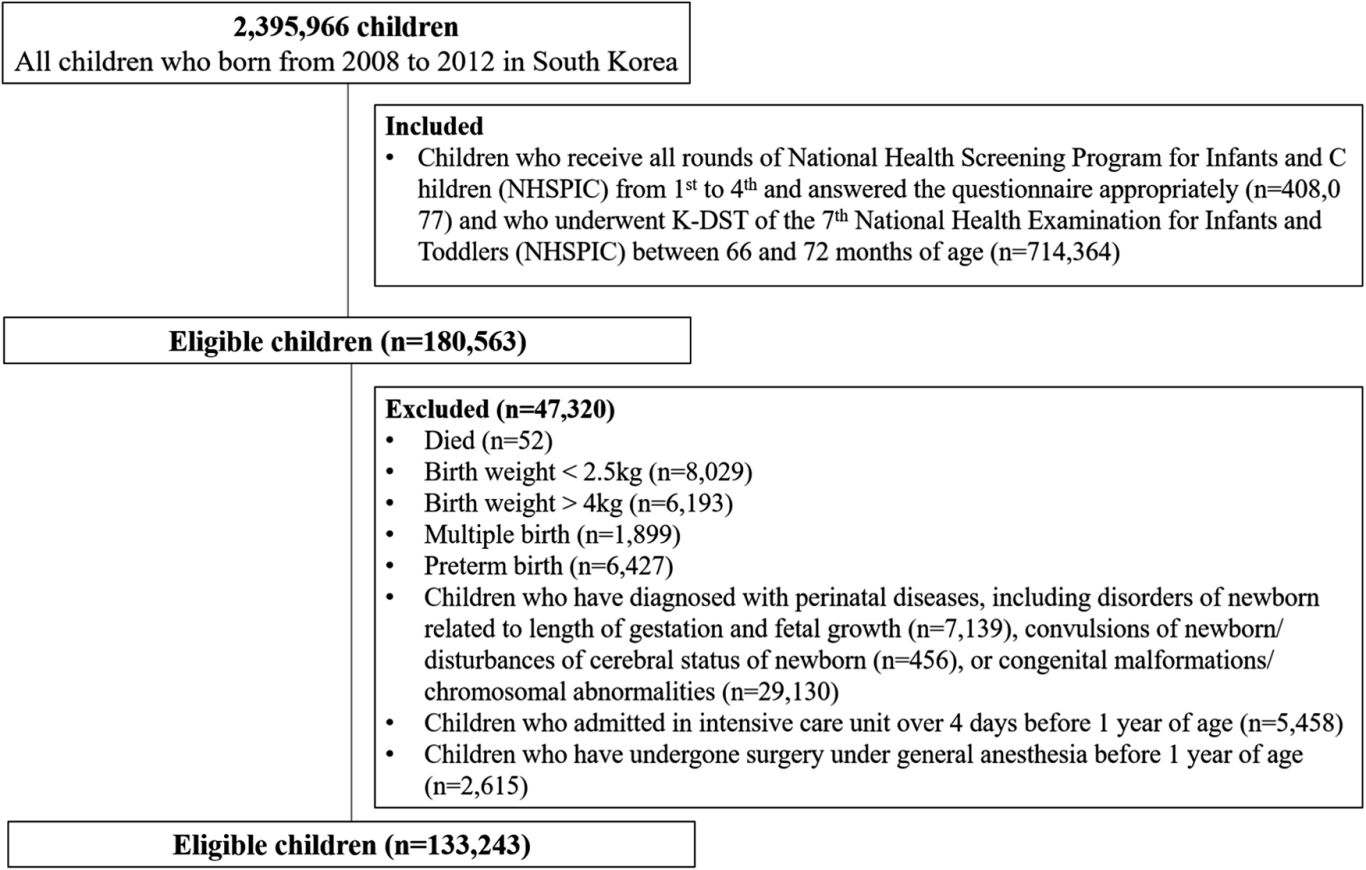
Study population.

The study population is shown in Fig. 1. We included out of the 2,395,966 Korean children born between 2008 and 2012 those children who received all rounds of the NHSPIC from the first to the fourth round, responded appropriately to the questionnaire (*n* = 408,077) and received the K-DST properly in 7th round (*n* = 714,364). In total, 180,563 children met the inclusion criteria. Subsequently, children who met the following criteria were excluded: (1) died (*n* = 52), (2) birth weight <2.5 kg (*n* = 8029) or >4 kg (*n* = 6193), (3) multiple births (*n* = 1899), (4) preterm birth (*n* = 6427), (5) diagnosis with disorders of newborn related to the length of gestation and fetal growth (*n* = 7139), convulsions of newborn/disturbances of cerebral status of newborn (*n* = 456), or congenital malformations/chromosomal abnormalities (*n* = 29,130), (6) admission in intensive care unit over 4 days before 1 year of age (*n* = 5458), and (7) who received general anesthesia before 1 year of age (*n* = 2615) and for >5 days before 2 years of age were excluded. Ultimately, we enrolled 133,243 eligible children.

### Dietary patterns in young childhood

The information on dietary patterns from infancy to 3 years of age was provided from the NHSPIC questionnaire, spanning the first through fourth rounds. The details of the questionnaire were described in Supplementary Table 1. Specifically, the first round of the NHSPIC, conducted at the age of 4−6 months, includes questions about the types of milk infants consume. The second round, conducted at the age of 9−12 months, includes questions about the initiation time of the introduction complementary foods, the frequency of intake of complementary foods, and the ingredients included in complementary foods. The third round, conducted at the age of 18−24 months, included questions about the consumption frequency of fruit juice or sweetened beverages. Finally, the fourth survey, conducted at the age of 30−36 months, includes questions about the frequency of fruit juice or sweetened beverage consumption, meal frequency, and milk intake.

### Clusters according to dietary patterns during young childhood

Polytomous Variable Latent Class Analysis (poLCA) was used to identify groups of similar cases within the manifest variables for dietary patterns during young childhood and determine whether they were statistically independent. We generated a series of models featuring a diverse range of latent clusters, spanning from two to ten. We evaluated the performance of each model to determine the optimal fit of the data and the greatest possible distinction between the identified clusters. We utilize several statistical measures to evaluate the quality of the model fit, including the maximum log-likelihood plot, which indicates the point at which the maximum log-likelihood ceases to increase significantly, and the elbow heuristic for the Bayesian Information Criterion (BIC) and Akaike Information Criterion (AIC), where the change in successive values becomes less noticeable. (Supplementary Table 3 and Supplementary Fig. 1) [14–18]. In addition, entropy values greater than 0.6 indicate good cluster separation [19, 20], and we considered the distribution of clusters acceptable when each cluster comprised more than 3% of the total participants. Based on the final model, four clusters were determined to provide the best fit.

### Developmental status at preschool age

The preschoolers’ developmental status was assessed using the K-DST performed at the age of 66–72 months, which is a valid screening tool designed specifically for Korean children and is part of the NHSPIC inventory [21, 22]. The K-DST consists of six domains: gross motor, fine motor, cognition, language, sociality, and self-care. Each domain consisted of eight questions answered by a parent or legal guardian, and the results were interpreted in four stages. These stages were: advanced development (total score ≤1 standard deviation [SD] score), age-appropriate (total score ≥–1 SD score and <1 SD score), need for follow-up (total score ≥–2 SD score and <–1 SD score), and recommendations for further evaluation (total score <–2 SD score). Children whose results indicated the need for follow-up underwent retesting or further evaluation if the interviews indicated problems. If the results for any of the six domains indicated the need for follow-up or recommendations for further evaluation, the total K-DST score was considered to reflect the same. The outcome of interest was an unfavorable outcome of K-DST, defined as a result of a “need for follow-up” or “recommendation for further evaluation” in each domain or total score.

### Covariates

Demographic variables such as sex, region at birth, economic status, and birth year were obtained from the NHIS database. The regions at birth were classified as Seoul, metropolitan, urban, or rural. Health insurance premiums were determined based on economic factors, including income level and assets. Therefore, the economic status was categorized into quintiles using health insurance premiums as the criteria for evaluation. Moreover, birth weight and head circumference at 4–6 months of age were considered baseline clinical variables and were obtained from the first round of the NHSPIC. In addition, diagnosed perinatal conditions, as baseline clinical variables, were observed using P-codes in ICD-10 codes. These condition included fetuses and newborns affected by maternal conditions, birth trauma, respiratory and cardiovascular disorders specific to the perinatal period, infections specific to the perinatal period, hemorrhagic and hematological disorders of the fetus and newborn, transitory endocrine and metabolic disorders specific to fetuses and newborns, digestive system disorders of the fetus and newborn, and conditions involving the integument and temperature regulation of the fetus and newborn. Furthermore, atopic dermatitis or food allergies, which can influence dietary habits, was assessed (details of disease definitions are provided in Supplementary Table 2).

### Statistical analysis

Categorical variables are expressed as the total number (n) and percentage (%), and continuous variables are described as mean and SD. Categorical variables between clusters were compared using the chi-square test, and continuous variables were compared using the Student’s *t* test. A multivariate logistic regression model was used to calculate odds ratios (ORs) with 95% confidence intervals (CIs) to identify the associations between dietary patterns and unfavorable K-DST outcomes. In addition, interaction *p* value between ORs were calculated by comparing the logarithmic differences of the ORs. The standard errors of these differences were used to derive Z-scores, from which *p* values were obtained to assess statistical significance. All analyses were adjusted for sex, region at birth, economic status, calendar year at birth, birth weight, head circumference at 4–6 months of age, perinatal conditions, and comorbidities. All the analyses were performed using the poLCA package (ver. 1.6.0.1) of R package (ver. 4.1.3) and SAS version 9.4 (SAS Institute Inc, Cary, NC, USA). Two-sided *P* < 0.05 was considered statistically significant.

## Results

### Characteristics of clusters according to dietary pattern during young childhood

Of 133,243 eligible children, the study identified four distinct clusters: control, which included 71,169 children (53.4%); cluster 1, which included 47,990 children (36.0%); cluster 2, which included 8750 children (6.6%); and cluster 3, which included 5334 children (4.0%). The dietary patterns associated with each cluster are presented in Table 1.

**Table 1:** Feeding characteristics according to type of clusters^a^.

Variables		*n* (%)				
Question	Answer	Total (*n* = 133,243)	Control (*n* = 71,169)	Cluster 1 (*n* = 47,990)	Cluster 2 (*n* = 8750)	Cluster 3 (*n* = 5334)
Types of feeding during the first 4 months of age^b^	Only human milk	62,969 (47.3)	36,744 (51.6)	22,089 (46.0)	1979 (22.6)	2157 (40.4)
	Only formula milk	42,375 (31.8)	19,465 (27.4)	15,774 (32.9)	4891 (55.9)	2245 (42.1)
	Mixed	27,899 (20.9)	14,960 (21.0)	10,127 (21.1)	1880 (21.5)	932 (17.5)
Timing of initiation of weaning food^c^	<4 months	3170 (2.4)	1460 (2.1)	1029 (2.1)	487 (5.6)	186 (3.5)
	4–6 months	104,966 (78.8)	61,034 (85.8)	35,615 (74.2)	5131 (58.6)	3186 (59.7)
	>6 months or not yet	25,107 (18.8)	8667 (12.2)	11,346 (23.6)	3132 (35.8)	1962 (36.8)
Frequency of weaning food intake per day at 1 year of age^c^	None	39 (0.0)	0 (0.0)	3 (0.0)	21 (0.2)	15 (0.3)
	1	2729 (2.0)	177 (0.2)	1149 (2.4)	619 (7.1)	784 (14.7)
	2	31,254 (23.5)	7239 (10.2)	17,093 (35.6)	5052 (57.7)	1870 (35.1)
	3	96,121 (72.1)	62,090 (87.2)	29,035 (60.5)	2515 (28.7)	2481 (46.5)
	≥4	3100 (2.3)	1663 (2.3)	710 (1.5)	543 (6.2)	184 (3.4)
Types of solid foods introduced at 1 year of age^c^	Grains	121,379 (91.1)	70,241 (98.7)	40,406 (84.2)	8069 (92.2)	2663 (49.9)
	Vegetables	129,072 (96.9)	71,084 (99.9)	47,295 (87.5)	8475 (96.9)	2218 (41.6)
	Fruits	102,173 (76.7)	66,861 (93.9)	25,581 (53.3)	8340 (95.3)	1391 (26.1)
	Eggs	85,341 (64.0)	66,123 (92.9)	10,780 (22.5)	7684 (87.8)	754 (14.1)
	Fish	83,840 (62.9)	63,676 (89.5)	11,829 (24.6)	7817 (89.3)	518 (9.7)
	Meat	125,694 (94.3)	70,972 (99.7)	45,923 (95.7)	8128 (92.9)	671 (12.6)
Amount of juice consumed at 2 year of age^d^	Less than 200 ml/day	124,504 (93.4)	68,153 (95.8)	45,127 (94.0)	6449 (73.7)	4775 (89.5)
	200≤ and <500 ml/day	8105 (6.1)	2907 (4.1)	2658 (5.5)	2054 (23.5)	486 (9.1)
	500 ml/day or over	634 (0.5)	109 (0.2)	205 (0.4)	247 (2.8)	73 (1.4)
Amount of juice consumed at 3 year of age^e^	Less than 200 ml/day	125,385 (94.1)	68,948 (96.9)	45,371 (94.5)	6234 (71.2)	4832 (90.6)
	200≤ and <500 ml/day	7057 (5.3)	1996 (2.8)	2267 (4.7)	2342 (26.8)	452 (8.5)
	500 ml/day or over	801 (0.6)	225 (0.3)	352 (0.7)	174 (2.0)	50 (0.9)
Frequency of meal intake per day at 3 year of age^e^	1	361 (0.3)	58 (0.1)	112 (0.2)	127 (1.5)	64 (1.2)
	2	12,015 (9.0)	2853 (4.0)	4910 (10.2)	3536 (40.4)	716 (13.4)
	3	119,388 (89.6)	67,867 (95.4)	42,564 (88.7)	4542 (51.9)	4415 (82.8)
	≥4	1479 (1.1)	391 (0.5)	404 (0.8)	545 (6.2)	139 (2.6)
Amount of milk consumed at 3 year of age^e^	None	31 (0.0)	7 (0.0)	21 (0.0)	2 (0.0)	1 (0.0)
	Less than 200 ml/day	46,536 (34.9)	25,311 (35.6)	16,903 (35.2)	2581 (29.5)	1741 (32.6)
	200≤ and <500 ml/day	79,473 (59.6)	43,771 (61.5)	28,38 (59.1)	4241 (48.5)	3075 (57.6)
	500≤ and <1000 ml/day	7003 (5.3)	2071 (2.9)	2623 (5.5)	1822 (20.8)	487 (9.1)
	1000 ml/day or over	200 (0.2)	9 (0.0)	57 (0.1)	104 (1.2)	30 (0.6)

*n* number.

^a^Children were classified based on their dietary patterns in infancy and young childhood using polytomous variable latent class analysis.

^b^This questionnaire was answered by their parents or legal guardians when their children were 4–6 months of age in the first round of NHSPIC.

^c^This questionnaire was answered by their parents or legal guardians when their children were 9–12 months of age in the second round of NHSPIC.

^d^This questionnaire was answered by their parents or legal guardians when their children were 18–24 months of age in the third round of NHSPIC.

^e^This questionnaire was answered by their parents or legal guardians when their children were 30–36 months of age in the fourth round of NHSPIC.

The control cluster had the highest prevalence of human milk in infancy (51.6%), started weaning food between four and six months of age (85.8%), and provided most of the suggested ingredients in weaning foods approximately three times daily. In addition, at 2–3 years of age, more than two meals were provided (95.9%), juice intake was low, and 61.5% of children consumed 200–500 ml of milk daily.

Second, the Cluster 1had a higher rate of weaning initiation after 6 months of age than the control cluster (23.6 vs 12.2%). The most notable distinction between Cluster 1 and the other clusters was children’s selective consumption of weaned food. While the majority of children in this group included consumed grains, vegetables, and meat in their weaning diet (84.2%, 87.5%, and 95.7%, respectively), a relatively small proportion consumed fruits, eggs, and fish (53.3%, 22.5%, and 24.6%, respectively). Juice intake at 2 and 3 years of age was similar to that of the control cluster, with 94% of the children consuming 200 ml or less daily. In addition, the frequency of eating at around three years of age was two to three times, which accounted for most of the time (98.9%).

Third, the Cluster 2 had the lowest prevalence of human milk (22.6%) and was characterized by a late initiation of weaning food after 6 months of age (35.8%) despite the use of a variety of ingredients (87.8–96.9% of children consumed any type of solid food at 1 years of age). Compared to the control cluster, the ratio of children who drink sweetened juice 200 ml per day or more was high (4.3 vs 26.3% at the age of 2 years, 3.1 vs 28.8% at 3 years). Furthermore, almost 40% of the children under the age of three years had fewer than three meals daily.

Finally, in Cluster 3, exclusively human milk was used at a prevalence rate of 40.4%. The highest proportion was observed for introducing complementary feeding after six months (36.8%) among the four clusters. In contrast, the lowest proportion was observed for introducing all ingredients in complementary foods until 12 months of age (9.7–49.9%). At age 3, 85.4% of children ate more than three times daily, and 57.6% consumed between 200 to 500 ml of milk daily. Approximately 10% of the children consumed more than 200 ml of juice at 2 and 3 years of age.

### Baseline characteristics of the study population

The baseline demographic and clinical characteristics of each cluster are presented in Table 2 and Supplementary Table 4. The study revealed that there were slightly more females than males in the control cluster (49.7% vs. 50.3%). The male-to-female ratios in the cluster 1 and 3 were similar to those in the control cluster. However, Cluster 2 showed the opposite trend, with a higher proportion of males than females (51.3 vs 48.7%, respectively). In addition, the four clusters differed in the region at birth and economic status (*P* < 0.01). The control cluster had a higher proportion of children born in Seoul and metropolitan areas and children in the highest quintile of economic status. In contrast, Cluster 3 had a higher proportion of children born in cities and rural areas and children from the lowest quintile of economic status.

**Table 2:** Baseline demographic and clinical characteristics according to cluster^a^.

	Total (*n* = 133,243)	Control (*n* = 71,169)	Cluster 1 (*n* = 47,990)	Cluster 2 (*n* = 8750)	Cluster 3 (*n* = 5334)
Sex, *n*(%)					
Boy	66,105 (49.6)	35,358 (49.7)	23,625 (49.2)	4490 (51.3)	2632 (49.3)
Girl	67,138 (50.4)	35,811 (50.3)	24,365 (50.8)	4260 (48.7)	2702 (50.7)
Regions at birth, *n*(%)					
Seoul	26,612 (20.1)	15,069 (21.3)	9017 (18.9)	1592 (18.4)	934 (17.7)
Metropolitan	33,113 (25.0)	17,373 (24.6)	12,314 (25.9)	2194 (25.3)	1232 (23.3)
City	64,345 (48.7)	34,042 (48.2)	23,384 (49.1)	4236 (48.9)	2683 (50.7)
Rural	8118 (6.1)	4146 (5.9)	2884 (6.1)	649 (7.5)	439 (8.3)
Economic status^b^, *n*(%)					
First quintile (lowest)	9163 (7.2)	4613 (6.8)	3242 (7.0)	789 (9.4)	519 (13.8)
Second quintile	17,643 (13.8)	8879 (13.1)	6373 (13.9)	1473 (17.5)	918 (24.4)
Third quintile	35,576 (27.9)	18,419 (27.1)	13,176 (28.6)	2462 (29.3)	1519 (40.4)
Fourth quintile	43,709 (34.3)	23,908 (35.2)	15,793 (34.3)	2508(29.9)	150 (4.0)
Fifth quintile (highest)	21,388 (16.8)	12,154 (17.9)	7417 (16.1)	1165 (13.9)	652 (17.3)
Calendar year at birth, *n*(%)					
2008–2009	39,595 (29.7)	17,635 (24.8)	17,465 (36.4)	2432 (27.8)	2063 (38.7)
2010–2012	93,648 (70.3)	53,535 (75.2)	30,525 (63.6)	6318 (72.2)	3271 (61.3)
Birth weight^c^, mean (SD), kg	3.22 (0.33)	3.22 (0.33)	3.22 (0.33)	3.22 (0.33)	3.21 (0.33)
Head circumference at 4–6 months of age^c^, mean (SD), cm	42.70 (1.46)	42.72 (1.45)	42.69 (1.46)	42.73 (1.48)	42.61 (1.50)

*n* number, *SD* standard deviation.

^a^Children were classified based on their dietary patterns in infancy and young childhood using polytomous variable latent class analysis.

^b^Socioeconomic status was determined according the amount of insurance co-payment, and stratified into quintiles.

^c^Measured or obtained by their parents or legal guardians when their children were 4–6 months of age in the first round of National Health Screening Program for Infants and Children.

The mean birth weight was 3.22 kg (SD, 0.33), with no significant differences among the groups, except for Cluster 3, which had a slightly lower mean birth weight (mean [SD], 3.21 [0.33]). In addition, there was no difference in head circumference at 4–6 months of age between the control cluster (mean [SD], 42.72 cm [1.45 cm]) and Cluster 2 (42.73 [1.48]), but cluster 1 and 3 (42.69 [1.46] and 42.61 [1.50], respectively) were slightly smaller than the those in the control cluster.

### The association between the dietary pattern during infancy and young childhood and developmental status at preschool age

**Fig. 2:**
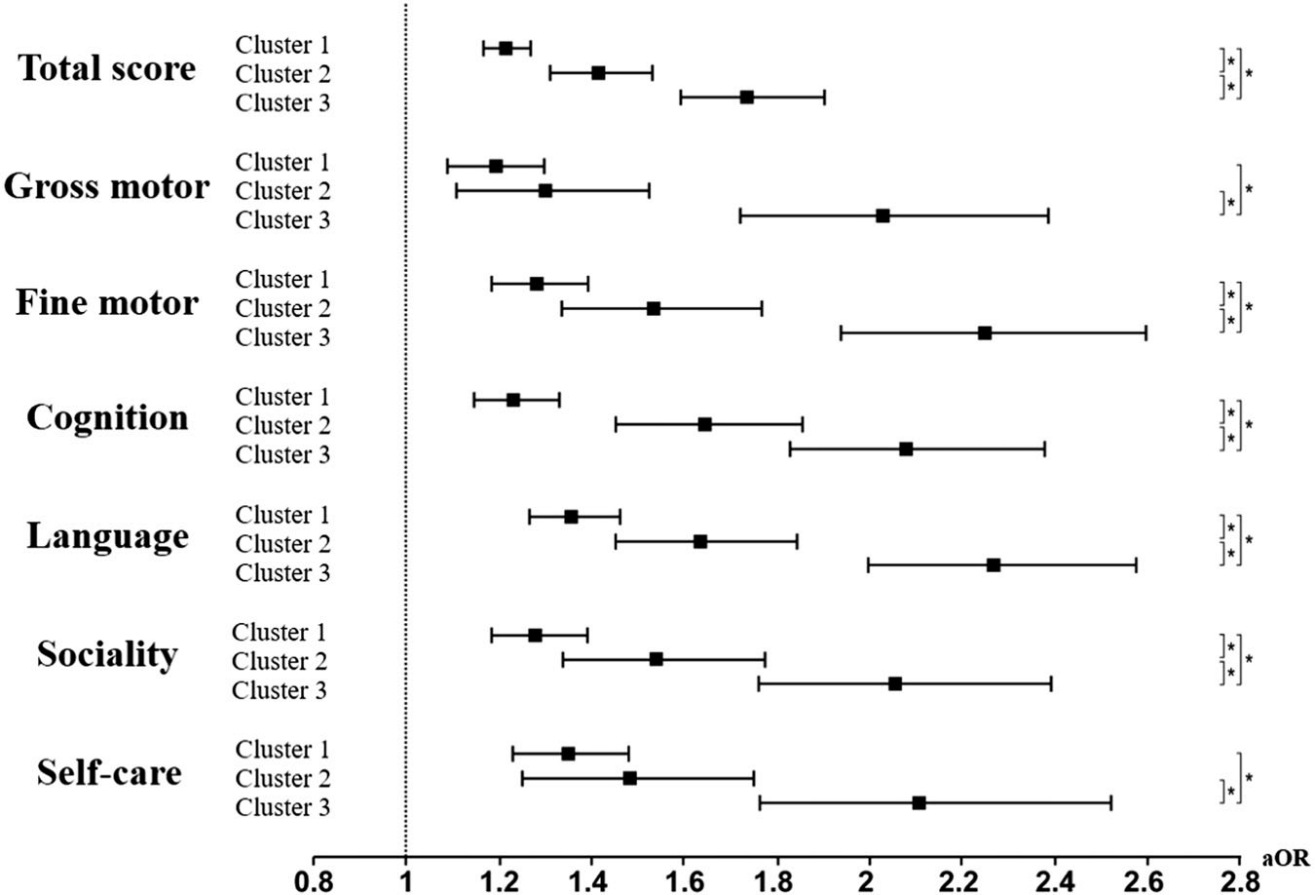
Diverse weaning foods and diet patterns at multiple time points during infancy period and their association with neurodevelopmental outcomes in 6-year-old children. Children were classified based on their dietary patterns in infancy and young childhood using polytomous variable latent class analysis. Adjusted odd ratios and their 95% confidence intervals were calculated by a multivariate logistic regression model with adjustment for sex, region at birth, economic status, birthweight, body weight at 4–6 and 9–12 months of age, head circumference at 4–6 months of age, perinatal conditions, and comorbidities. Filled squares indicate aOR and black lines indicate 95% CI. An asterisk indicates that the interaction *p*-value between two odds ratios is less than 0.05. aOR adjusted odd ratio.

The associations between dietary patterns and developmental screening test results are shown in Table 3 and Fig. 2. Considering the control cluster as a reference, the results of the K-DST performed at six years of age were compared with those of the other clusters. The number of children with unfavorable outcomes according to the total score was 4448 (6.2%) in the control cluster, 3592 (7.5%) in Cluster 1, 794 (9.1%) in Cluster 2, and 586 (11.0%) in Cluster 3. The aOR for the unfavorable outcome of the total score was 1.209 (95% CI, 1.156–1.266) for Cluster 1, 1.418 (95% CI, 1.312–1.532) for Cluster 2, and 1.741 (95% CI, 1.593–1.903) for Cluster 3. This indicates that, compared to the control cluster, the impact on abnormal K-DST results at preschool age increases progressively from Cluster 1 to Cluster 2 and is strongest in Cluster 3 (all interaction *p*-values < 0.05).

**Table 3:** Associations of poor outcomes on the K-DST at 6 years of age in each cluster compared with the healthy diet cluster^a^.

	Poor outcome of K-DST^b^						
	Control (*n* = 71,169)	Cluster 1 (*n* = 47,990)		Cluster 2 (*n* = 8750)		Cluster 3 (*n* = 5334)	
	*n*(%)	*n*(%)	aOR (95% CI)	*n*(%)	aOR (95% CI)	*n*(%)	aOR (95% CI)
Total score	4448 (6.2)	3,596 (7.5)	1.209 (1.156–1.266)	794 (9.1)	1.418 (1.312–1.532)	586 (11.0)	1.741 (1.593–1.903)
Domain							
Gross motor	1149 (1.6)	922 (1.9)	1.186 (1.084–1.297)	189 (2.2)	1.300 (1.108–1.524)	176 (3.3)	2.030 (1.724–2.389)
Fine motor	1275 (1.8)	1093 (2.3)	1.279 (1.176–1.390)	255 (2.9)	1.535 (1.336–1.764)	223 (4.2)	2.248 (1.940–2.605)
Cognition	1666 (2.3)	1370 (2.9)	1.231 (1.143–1.326)	353 (4.0)	1.644 (1.459–1.851)	270 (5.1)	2.085 (1.825–2.382)
Language	1646 (2.3)	1530 (3.2)	1.355 (1.260–1.457)	345 (3.9)	1.634 (1.450–1.841)	293 (5.5)	2.270 (1.997–2.580)
Sociality	1264 (1.8)	1108 (2.3)	1.279 (1.176–1.391)	245 (2.8)	1.541 (1.338–1.775)	203 (3.8)	2.055 (1.762–2.396)
Self-care	942 (1.3)	845 (1.8)	1.345 (1.221–1.482)	175 (2.0)	1.482 (1.254–1.752)	144 (2.7)	2.111 (1.765–2.526)

*K-DST* Korean developmental screening test, *n* number, *aOR* adjusted odd ratio, *CI* confidence interval.

^a^Children were classified based on their dietary patterns in infancy and young childhood using polytomous variable latent class analysis.

^b^A poor outcome of the K-DST was defined as need for follow-up or recommendation for further evaluation.

^c^Adjusted hazard ratios and their 95% confidence intervals were calculated using a multivariate logistic regression with adjustments for sex, region at birth, economic status, birthweight, body weight at 4–6 and 9–12 months of age, head circumference at 4–6 months of age, perinatal conditions, and comorbidities.

Additionally, the associations between unfavorable outcomes and individual domains of the K-DST were consistent with the total score. Compared to the control cluster, the associations with unfavorable outcomes were significant across all individual domains for the other clusters, with Cluster 3 showing more than twice the strength of association with unfavorable outcomes compared to the control cluster.

## Discussion

This national administrative and observational cohort study evaluated neurodevelopment at age 6 across various domains by categorizing children into four groups based on their dietary patterns during infancy and early childhood. Compared to a control cluster, the risk of unfavorable results in the neurodevelopmental screening test increased in the following order: Cluster 1, which involved selective ingredient use at at age 1; Cluster 2, characterized by high juice consumption; and Cluster 3, with a limited variety of ingredients at 1 year of age. It was confirmed that these differences in neurodevelopment could be applied to all domains of developmental screening tests, including gross motor, fine motor, cognition, language, sociality, and self-care. This study confirmed that dietary patterns in infants and young children are associated with neurodevelopment at 6 years of age.

The recommended dietary guidelines for infants and young children are as follows: Human milk feeding is encouraged during infancy [23], with the introduction of weaning foods typically beginning between 4 to 6 months of age [24]. Additionally, both infants and toddlers should be offered three main meals composed of a variety of ingredients [25, 26], along with a recommended milk intake of 200–400 ml [25, 27, 28]. The control cluster aligns well with these recommendations, characterized by a high rate of human milk consumption, the introduction of weaning foods between 4–6 months, a diverse range of food ingredients, and an appropriate meal frequency.

Compared to the control cluster, Cluster 1, 2, and 3 had a higher risk of abnormal outcomes of neurodevelopmental screening test by the K-DST at preschool age. Cluster 1 had slightly lower prevalence of human milk in infancy and a slightly higher proportion of children who initiated weaning food after 6 months. The most distinctive feature of Cluster 1 was picky eating regarding meal ingredients. While most children in this cluster were introduced to grains, vegetables, and meat, only a small proportion consumed fruit, eggs, and fish. In addition, Cluster 2 had the lowest prevalence of human milk consumption during infancy and the lowest proportion of children who ate three times a day at 1 and 3 years of age. Furthermore, Cluster 2 had the largest number of children among the four groups who consumed more than 200 ml of juice at ages 2 and 3. Cluster 3 had the highest proportion of children who initiated weaning after 6 months. The most distinctive feature of Cluster 3 was that many children were not introduced to ingredients such as fish, meat, eggs, or fruit in their diet at 1 year of age. The rate of consumption of grains or vegetables was less than half.

There are several potential mechanisms by which children with dietary patterns that deviate from the recommended guidelines in infancy and young childhood may experience negative impacts on neurodevelopment. First, the observed effects on neurodevelopmental outcomes may be linked to the role of an enriched environment, including feeding practices. The typical approach to implementing enriched environmental resources involves incorporating sensory and motor stimulation using various nest materials [29]. Animal experiments have demonstrated the positive effects of enriched environments on neurodevelopment [30, 31]. In addition, enriched resources have recently been recognized as relevant in the context of brain disorders [29, 32]. Additionally, enriched environments treat various developmental conditions in children, such as autism [33]. Sensory integration therapy typically involves visual, auditory, and tactile stimuli. Appropriate timing and various kinds of introduction of weaning food will be able to give these various gustatory stimuli, and it can be seen that this mechanism could be one of the important mechanisms in which the introduction of weaning food correlates with the development.

Second, Previous studies have investigated the impact of food on neurodevelopment from various perspectives, including nutrition [34, 35]. Assorted weaning food offers essential nutrients, including vitamins and minerals, crucial for physical growth and neurodevelopment. Studies have shown that growth during infancy, particularly in height and length, is associated with verbal and performance IQ in school-aged children [36]. Additionally, lower length measurements at 4 and 12 months were linked to lower cognitive function scores at 24 months [37]. As infants grow, their energy and nutrient requirements increase, necessitating additional nutrients such as iron, zinc, iodine, and various cofactors [38]. Micronutrient and caloric supplementation from complementary foods, especially in children of low socioeconomic status, promotes physical growth and neurodevelopment [10, 39]. The early growth patterns facilitated by introducing diverse weaning foods may contribute to improved brain development.

Last, the gut microbiome, influenced by diet and nutrition in children, significantly modulate brain function [40, 41]. Diet pattern influences gut microbiome via macronutrients and bioactive molecules and it also influences children’s attention [42]. In imaging studies, microbiome composition in infants affects early learning and cognitive scores at 2 years old without the differences in brain volume [43]. A longitudinal cohort study reported that specific microbiome patterns, dominantly including Genus *Bacteroides* were correlated with cognitive and language scores at children’s age 2 [44]. Children with autism spectrum disorder and cognitive and communication impairment have a specific pattern of microbiome and human transcriptome in age- and sex-matched cohort [45]. The body of evidence supporting the connection between the gut microbiome and neurodevelopment in early infancy and the relationship between the microbiome and brain development continues to expand.

This study had several notable strengths. First, it employed a longitudinal design with a large, representative sample of children, allowing for robust and generalizable findings. Second, poLCA use facilitated efficient observation of the effects of dietary patterns at different time points on children’s development through classification [46]. Thirdly, in contrast to previous research that primarily emphasized the benefits of human or fortified milk on children’s neurodevelopment, this study specifically focused on examining the impact of various dietary patterns during infancy and young childhood.

It is important to acknowledge the limitations of this study. First, information on dietary patterns was obtained from caregiver recall surveys, which introduced a potential recall bias. However, it is considered reliable because the survey asks about dietary patterns at various times. Additionally, it is challenging to ascertain comprehensive developmental outcomes based solely on the results of the K-DST performed at a single time point. Children exhibit individual variations in their developmental rates, and even those initially suspected of experiencing developmental demonstrate normal later often. Therefore, caution should be exercised when interpreting these results. Furthermore, disparities in the educational attainment of children and parents may exist despite the government’s provision of subsidies for preschool daycare centers. Moreover, factors related to the growth environment, such as siblings, family, and community, were not analyzed. These differences could be confounding variables; unfortunately, our dataset did not include this information.

In conclusion, we suggest that dietary practices during infancy and young childhood, including feeding type, timing of weaning food introduction, ingredients used in foods, frequency of intake, and the consumption of juice and milk, may influence neurodevelopment in preschool-aged children.

## Supplementary information

supplementary tables

## Data Availability

This study was based on the National Health Claims Database established by the National Health Insurance Service of the Republic of Korea. Applications for using the National Health Insurance Service data are reviewed by the Inquiry Committee of Research Support; if the application is approved, raw data are provided to the applicant for a fee. We cannot provide access to the data, analytic methods, and research materials to other researchers because of the intellectual property rights of this database that is owned by the National Health Insurance Corporation. However, investigators who wish to reproduce our results or replicate the procedure can use the database, which is open for research purposes (https://nhiss.nhis.or.kr/ accessed on 7 July 2023).
