# Supplementary material for: Diverse weaning foods and diet patterns at multiple time points during infancy period and their association with neurodevelopmental outcomes in 6-year-old children
Source: Eur J Clin Nutr. 2024 Oct 18;79(2):168–75. doi: 10.1038/s41430-024-01528-3 (PMC11810780; doi:10.1038/s41430-024-01528-3)

**Supplementary table 1. Questionnaire for dietary pattern.**

| **Round of the NHSPIC** | **Age (months)** | **Question** | **Answer** |
| --- | --- | --- | --- |
| 1^st^ | 4 – 6 | Which type of milk do you typically give to your child? | Please select only one of the following options:  (1) Only breastmilk  (2) Only formula milk  (3) A mixture of breastmilk and formula milk  (4) Special milk powder |
| 2^nd^ | 9 – 12 | When did your child start weaning? | Please select only one of the following options:  (1) < 4 months  (2) 4 – 6 months  (3) > 6 months or not yet |
|  |  | How many times a day do you provide weaning food to your child? | Please select only one of the following options:  (1) none  (2) 1  (3) 2  (4) 3  (5) 4 or over |
|  |  | Do weaning foods contain the following ingredients?  (1) grains  (2) vegetables  (3) fruits  (4) eggs  (5) fish  (6) meat | Choose one answer for each item:  (1) yes  (2) no |
| 3^rd^ | 18 – 24 | How much fruit juice or sweetened beverages does your child drink per day? | Please select only one of the following options:  (1) less than 200ml  (2) 200≤ and <500ml  (3) 500ml or over |
| 4^th^ | 30 – 36 | How much fruit juice or sweetened beverages does your child drink per day? | Please select only one of the following options:  (1) less than 200ml  (2) 200≤ and <500ml  (3) 500ml or over |
|  |  | How many meals a day does your child eat? | Please select only one of the following options:  (1) 1  (2) 2  (3) 3  (4) 4 or over |
|  |  | How much milk does your child drink per day? | Please select only one of the following options:  (1) less than 200ml  (2) 200≤ and <500ml  (3) 500≤ and <1,000ml  (4) 1,000ml or over |

**Supplementary table 2. The definitions of perinatal comorbidities**

| **Diseases** | **Definitions** |
| --- | --- |
| Perinatal comorbidities |  |
| Fetus and newborn affected by maternal conditions | Diagnosis of ICD-10 code P00.X – P04.X |
| Birth trauma | Diagnosis of ICD-10 code P10.X – P15.X |
| Respiratory and cardiovascular disorders specific to the perinatal period | Diagnosis of ICD-10 code P20.X – P29.X |
| Infections specific to the perinatal period | Diagnosis of ICD-10 code P35.X – P39.X |
| Haemorrhagic and haematological disorders of fetus and newborn | Diagnosis of ICD-10 code P50.X – P61.X |
| Transitory endocrine and metabolic disorders specific to fetus and newborn | Diagnosis of ICD-10 code P70.X – P74.X |
| Digestive system disorders of fetus and newborn | Diagnosis of ICD-10 code P75.X – P78.X |
| Conditions involving the integument and temperature regulation of fetus and newborn | Diagnosis of ICD-10 code P80.X – P83.X |
| Congenital malformations, deformations and Other disorders originating in the perinatal period | Diagnosis of ICD-10 code P90.X – P96.X |
| Comorbidity |  |
| Atopic dermatitis | Diagnosis of ICD-10 code L20.X before 36 months of age |
| Food allergy | Diagnosis of ICD-10 code L23.6, L24.6, L25.4, L27.2, T78.0, T78.1, or K52.2 before 36 months of age |

**Supplementary table 3. Results of proLCA function**

| Number of clusters | AIC | BIC | G2 | X2 | n of estimated parameters | residual degrees of freedom | maximum log likelihood | entropy |
| --- | --- | --- | --- | --- | --- | --- | --- | --- |
| 2 | 1696492 | 1696992 | 50395.06 | 24224014 | 51 | 133192 | -848194 | 0.428 |
| 3 | 1686325 | 1687080 | 46483.59 | 24675260 | 77 | 133166 | -846239 | 0.526 |
| 4 | 1682546 | 1683555 | 36032.44 | 1770263 | 103 | 133140 | -841013 | 0.601 |
| 5 | 1679692 | 1680957 | 33439.71 | 1536864 | 129 | 133114 | -839717 | 0.656 |
| 6 | 1679434 | 1680953 | 31981.3 | 1255417 | 155 | 133088 | -838987 | 0.707 |
| 7 | 1677024 | 1678798 | 30667.41 | 1144554 | 181 | 133062 | -838331 | 0.766 |
| 8 | 1676494 | 1678525 | 30088.16 | 1125664 | 207 | 133036 | -838037 | 0.805 |
| 9 | 1675961 | 1678245 | 29500.54 | 1163147 | 233 | 133010 | -837804 | 0.840 |
| 10 | 1675764 | 1678302 | 29251.3 | 927236 | 259 | 132984 | -837622 | 0.902 |

Abbreviation, LCA, latent cluster analysis; AIC, Akaike information criterion; BIC, Bayesian information criterion; G2, Likelihood ratio/deviance statistic; X2, the Chi-square statistic.

**Supplementary table 4. Perinatal comorbidities in participants**

| Characteristics | n (%) | | | | | P value | | | |
| --- | --- | --- | --- | --- | --- | --- | --- | --- | --- |
|  | Total  (133,243) | Healthy  diet  (n=71,169)^1^ | Imbalanced  diet  (n=47,990)^2^ | High juice consumption  (n=8,750)^3^ | Low variety diet  (n=5,334)^4^ | Overall | 1 vs 2 | 1 vs 3 | 1 vs 4 |
| Sex, n (%) |  |  |  |  |  | <0.01 | 0.13 | <0.01 | 0.63 |
| Boy | 66,105 (49.6) | 35,358 (49.7) | 23,625 (49.2) | 4,490 (51.3) | 2,632 (49.3) |  |  |  |  |
| Girl | 67,138 (50.4) | 35,811 (50.3) | 24,365 (50.8) | 4,260 (48.7) | 2,702 (50.7) |  |  |  |  |
| Regions at birth, n (%) |  |  |  |  |  | <0.01 | <0.01 | <0.01 | <0.01 |
| Seoul | 26,612 (20.1) | 15,069 (21.3) | 9,017 (18.9) | 1,592 (18.4) | 934 (17.7) |  |  |  |  |
| Metropolitan | 33,113 (25.0) | 17,373 (24.6) | 12,314 (25.9) | 2,194 (25.3) | 1,232 (23.3) |  |  |  |  |
| City | 64,345 (48.7) | 34,042 (48.2) | 23,384 (49.1) | 4,236 (48.9) | 2,683 (50.7) |  |  |  |  |
| Rural | 8,118 (6.1) | 4,146 (5.9) | 2,884 (6.1) | 649 (7.5) | 439 (8.3) |  |  |  |  |
| Economic status^2^, n (%) |  |  |  |  |  | <0.01 | <0.01 | <0.01 | <0.01 |
| First quintile (lowest) | 9,163 (7.2) | 4,613 (6.8) | 3,242 (7.0) | 789 (9.4) | 519 (13.8) |  |  |  |  |
| Second quintile | 17,643 (13.8) | 8,879 (13.1) | 6,373 (13.9) | 1,473 (17.5) | 918 (24.4) |  |  |  |  |
| Third quintile | 35,576 (27.9) | 18,419 (27.1) | 13,176 (28.6) | 2,462 (29.3) | 1,519 (40.4) |  |  |  |  |
| Fourth quintile | 43,709 (34.3) | 23,908 (35.2) | 15,793 (34.3) | 2,508(29.9) | 150 (4.0) |  |  |  |  |
| Fifth quintile (highest) | 21,388 (16.8) | 12,154 (17.9) | 7,417 (16.1) | 1,165 (13.9) | 652 (17.3) |  |  |  |  |
| Calendar year at birth, n(%) |  |  |  |  |  | <0.01 | <0.01 | <0.01 | <0.01 |
| 2008 – 2009 | 39,595 (29.7) | 1,7635 (24.8) | 17,465 (36.4) | 2,432 (27.8) | 2,063 (38.7) |  |  |  |  |
| 2010 – 2012 | 93,648 (70.3) | 53,535 (75.2) | 30,525 (63.6) | 6,318 (72.2) | 3,271 (61.3) |  |  |  |  |
| Birth weight^3^, mean (SD), kg | 3.22 (0.33) | 3.22 (0.33) | 3.22 (0.33) | 3.22 (0.33) | 3.21 (0.33) | <0.01 | 0.26 | 0.44 | <0.01 |
| Head circumference at 4 – 6 months  of age^3^, mean (SD), cm | 42.7 (1.46) | 42.72 (1.45) | 42.69 (1.46) | 42.73 (1.48) | 42.61 (1.50) | <0.01 | <0.01 | 0.54 | <0.01 |
| Perinatal comorbidities |  |  |  |  |  |  |  |  |  |
| Fetus and newborn affected by maternal conditions | 2,987 (2.2) | 1,632 (2.3) | 1,073 (2.2) | 181 (2.1) | 101 (1.9) | 0.17 | 0.52 | 0.18 | 0.06 |
| Birth trauma | 1,348 (1.0) | 777 (1.1) | 419 (0.9) | 102 (1.2) | 50 (0.9) | <0.01 | <0.01 | 0.53 | 0.29 |
| Respiratory and cardiovascular disorders  specific to the perinatal period | 6,913 (5.2) | 3,747 (5.3) | 2,458 (5.1) | 453 (5.2) | 255 (4.8) | 0.38 | 0.28 | 0.72 | 0.12 |
| Infections specific to the perinatal period | 17,600 (13.2) | 9,167 (12.9) | 6,554 (13.7) | 1,147 (13.1) | 732 (13.7) | <0.01 | <0.01 | 0.54 | 0.08 |
| Haemorrhagic and haematological disorders of fetus and newborn | 43,538 (32.7) | 23,283 (32.7) | 15,771 (32.9) | 2,771 (31.7) | 1,713 (32.1) | 0.13 | 0.59 | 0.05 | 0.37 |
| Transitory endocrine and metabolic disorders specific to fetus and newborn | 4,045 (3) | 2,297 (3.2) | 1,345 (2.8) | 242 (2.8) | 161 (3.0) | <0.01 | <0.01 | 0.02 | 0.40 |
| Digestive system disorders of fetus and newborn | 3,150 (2.4) | 1,605 (2.3) | 1,187 (2.5) | 224 (2.6) | 134 (2.5) | 0.04 | 0.01 | 0.07 | 0.22 |
| Conditions involving the integument and temperature regulation of fetus and newborn | 5,582 (4.2) | 2,853 (4.0) | 2,130 (4.4) | 368 (4.2) | 231 (4.3) | <0.01 | <0.01 | 0.38 | 0.24 |
| Congenital malformations, deformations and Other disorders originating in the perinatal period | 7,891 (5.9) | 4,118 (5.8) | 2,929 (6.1) | 513 (5.9) | 331 (6.2) | 0.11 | 0.02 | 0.77 | 0.21 |
| Comorbidity |  |  |  |  |  |  |  |  |  |
| Atopic dermatitis | 61,426 (46.1) | 31,746 (44.6) | 23,256 (48.5) | 3,935 (45.0) | 2,489 (46.7) | <0.01 | <0.01 | <0.01 | 0.52 |
| Food allergy | 1,100 (0.8) | 530 (0.7) | 462 (1.0) | 62 (0.7) | 46 (0.9) | <0.01 | <0.01 | 0.34 | 0.71 |

**Supplemenatary figure 1. Plot of Akaike information criterion, Bayesian information criterion, and maximum log likelihood.**


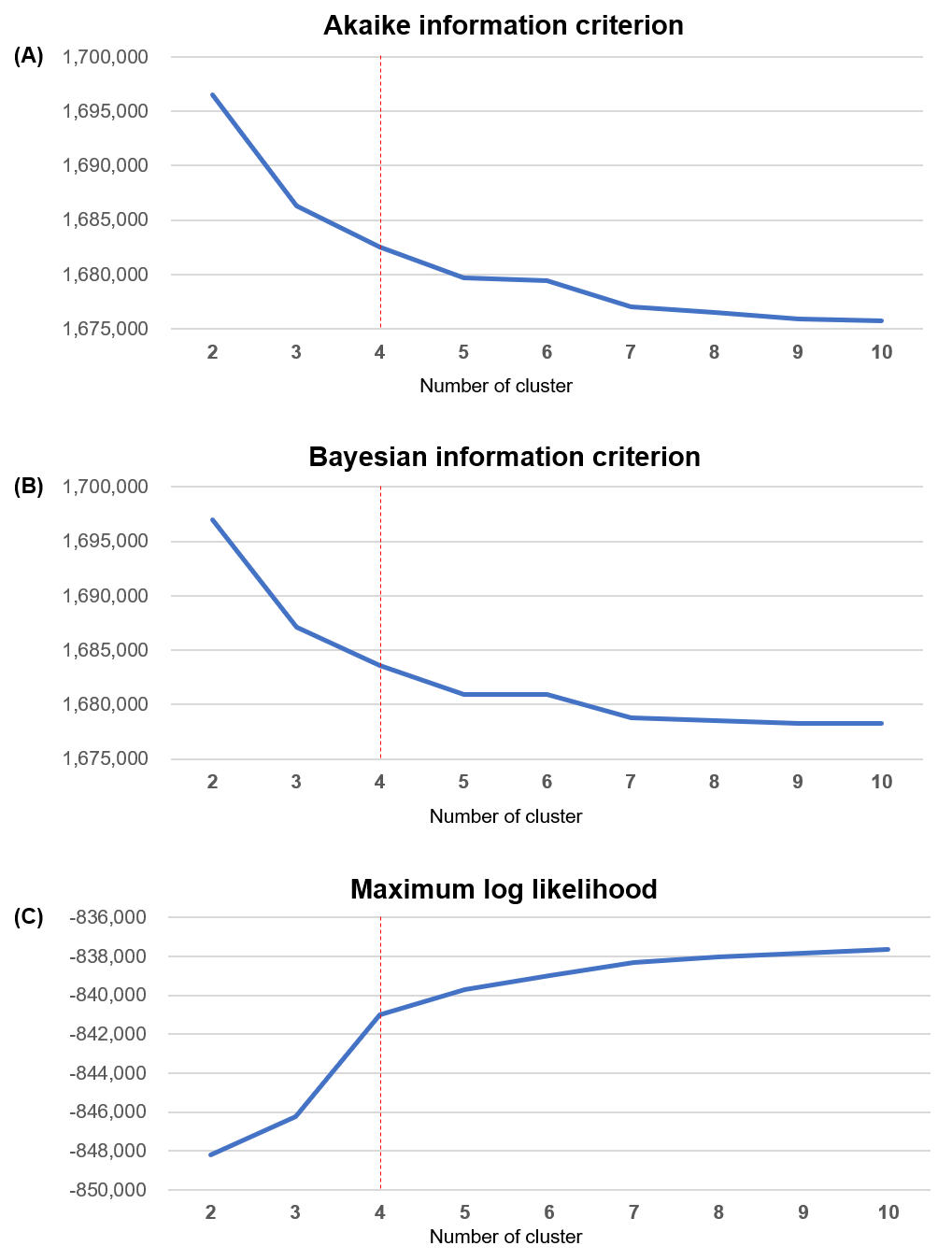

Supplement: Supplementary file 1 — supplementary tables [file 41430_2024_1528_MOESM1_ESM.docx]
